# Low energy consumption fiber-type memristor array with integrated sensing-memory†

**DOI:** 10.1039/d1na00703c

**Published:** 2022-01-18

**Authors:** Yanfang Meng, Jiaxue Zhu

**Affiliations:** Key Laboratory of Advanced Optical Communication Systems and Network, School of Electronic Engineering and Computer Science Department, Peking University Beijing 100091 China; Department of Engineering Mechanics, Tsinghua University Beijing 100084 China; Key Laboratory of Microelectronic Devices and Integrated Technology, Institute of Microelectronics of the Chinese Academy of Sciences Beitucheng West Road Beijing 100029 China

## Abstract

The increasing growth of electronic information science and technology has triggered the renaissance of the artificial sensory nervous system (SNS), which can emulate the response of organisms towards external stimuli with high efficiency. In traditional SNS, the sensor units and the memory units are separated, and therefore difficult to miniaturize and integrate. Here, we have incorporated the sensor unit and the memory unit into one system, taking advantage of the unique properties of the ion-gel system. Meanwhile, the weaving-type memory array presents paramount advantages of integration and miniaturization and conformal lamination to curved surfaces. It is worth noting that the electrical double layer (EDL) within the ion gel endow the device with a low operation voltage (<1 V) to achieve low energy consumption. Finally, according to the relationship of pressure stimuli and electrical behavior, the integrated responsiveness-storage external stimuli ability is achieved. Our work offers a new platform for designing cutting edge SNS.

## Abstract 2

To develop an artificial sensory nervous system, we use an ion-gel system and solve the issue that sensor units and memory units are separated and difficult to miniaturize and integrate. Consequently integrated responsiveness-storage external stimuli ability is achieved.

## Graphical abstract

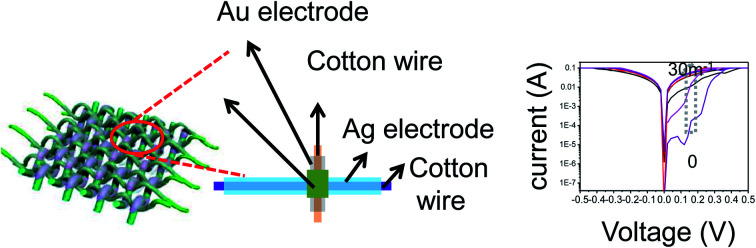

## 1 Introduction

Resistive switching-based memristors (RS), acting as a fascinating emerging storage technology, have received much attention due to their^1–8^ advantages, including feasible, fast operating speed, long time retention, high density, and low energy consumption. To mimic the functions of organisms for reacting to external stimuli and storing the sensed signal, integrated memory-sensing systems have sprung up. For example, Bowen Zhu *et al.*^9^ have integrated a pressure sensor into a memory device to establish a bridge between the state-of-the-art E-skin devices and skin-inspired-integration haptic memory devices to mimic the haptic memory of natural skin. In another instance, with respect to visual-memory systems, integrating UV light sensors onto the memristor array was capable of affording an essential exteroceptive sensory memory for cognitive tasks.^10,11^

Although these works may represent significant steps towards the realization of bio-inspired SNS, in these previous memory-sensing systems, the memory units and sensing units were independent and were not beneficial for the integration and miniaturization. Incorporating the memory units and sensing units into one system involves material selection, matched operation conditions and so on.

To solve the above issues, we present a memory-array based iontronic system, taking advantage of the stimuli-responsive characteristic of ions within this iontronic system. Specifically speaking, the iontronic can play a dual role: inductive stimulus and storing charges (as an insulator layer of a memristor). Meanwhile, for biomedical applications, such as implanted treatment and human–machine interface, it is highly desired to explore devices with excellent flexibility and mechanical compatibility with biological tissues.^12–14^ As a consequence, stretchable memristors have emerged to address the issue of implantable and comformal applications. With the springing up of fiber-based electronics and wearable electronic systems, functional fibers provide a promising platform for flexible and wearable memristors for computing and storage applications.^15,16^

Anjae Jo has reported a textile resistance switching memory based on Al-coated threads with a native layer of Al_2_O_3_ as a resistance switching layer and carbon fiber as the counter-electrode. However, the driving voltages of their devices are not relatively low, and the ON/OFF ratio was not high (∼10^3^). To alleviate the high energy consumption from a high operating voltage, the iontronics have been newly emerging, with the interdisciplinary concept that bridges electronics and ionics. Within the iontronics systems, the EDLT can realize the low-voltage operation to promote energy efficiency.^17–19^ The ionic systems utilized in electronics have been reported. Guoyun Gao *et al.* have developed a triboiontronic transistor of MoS_2_ with electric double layers (EDLs) formed in electrolyte-gated field-effect transistors (FETs), inducing an extremely large local electric field that gave a highly efficient charge carrier control in the semiconductor channel.^20^ The resistance random-access memory (ReRAM) could also be adopted iontronics, in which electrical switching effects between the on and off states are triggered by ionic motion in the oxide devices.^21^

In this manuscript, we, for the first time, have integrated the iontronic system into a wearable memristor electronic system to render the integration of stimuli-responsiveness-memory. The low-voltage operation has been achieved by the EDLT in ion-gel to considerably decrease energy consumption. In particular, we introduce the brain-inspired device technologies into the non-volatile resistive memory array. The device shows bidirectional continuous weight modulation behaviour. These experimental results consolidate the feasibility of sensing-memory integrated systems and pave a new avenue for constructing energy efficient and large-scale neuromorphic systems.

## 2 Results and discussion

### 2.1 Basic structure and fabrication procedure of the memristor

**Fig. 1:**
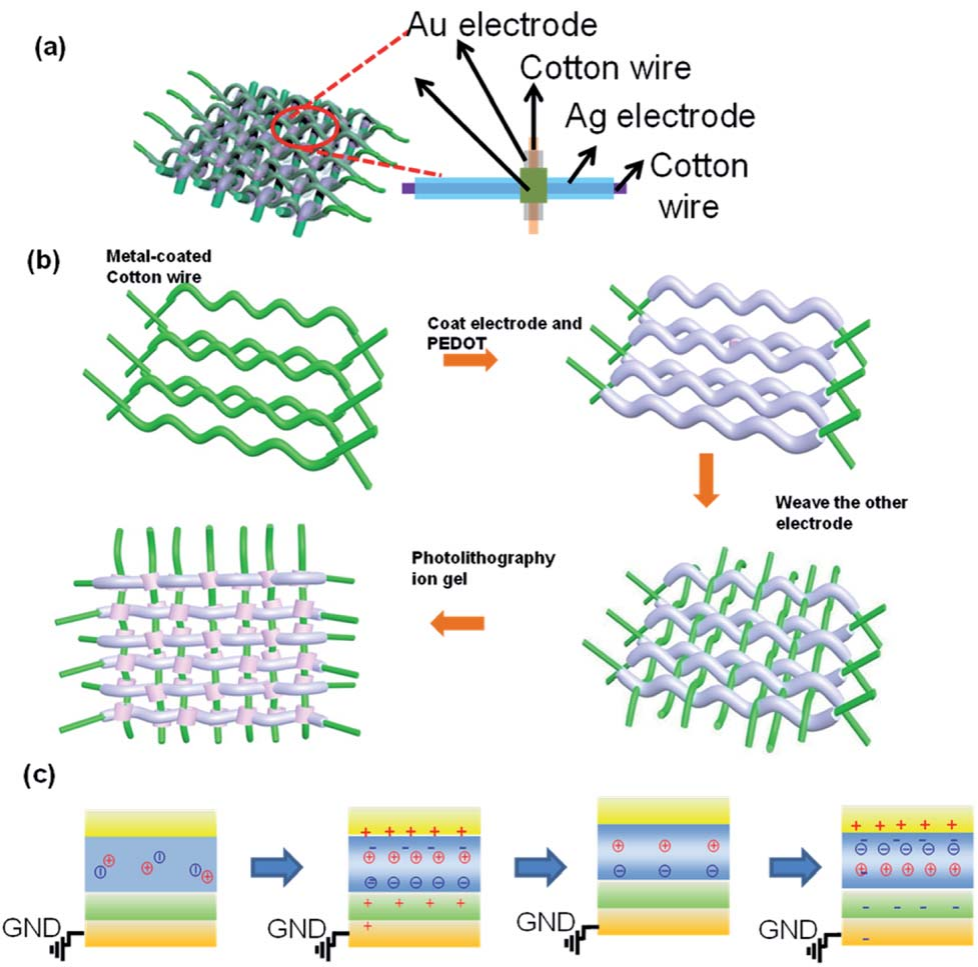
(a) Schematic illustration of an integrated memory-sensing system memristor array. (b) Fabrication process of the integrated memory-sensing system memristor array. (c) The mechanism of the fiber-type iontronic memristor.

Fig. 1a systematically illustrates the basic structure of the fiber-type memristor array with a magnified schematic diagram of a single unit of the array inserted. The wearable fiber-type memristor array is an electrode-insulator-electrode structure with Pt-coated cotton fiber and Ag-coated cotton fiber acting as inert electrode and active electrode, respectively. MIM configuration is a well-known structure for resistive switching devices when the inserted insulating materials have specific switching resistivity properties under the applied bias.^22,23^

The Ag-coated cotton fibers and Pt-coated cotton fibers are knitted with a vertical-horizontal cross configuration and the semiconductor and dielectric layer is sandwiched between them (as shown in Fig. 1a). The procedure for fabricating this memristor array is illustrated in Fig. 1b. Prior to being dipped-coated with PEDOT:PSS ink (1 wt%), the commercially-available cotton fiber in horizontal orientation selectively coated the inert electrode (*e.g.*, Pt, Au electrode) by magnetron sputtering. Then, the ion gel (composed of 1-ethyl-3-methylimidazolium bis(trifluoromethyl sulfonyl)imide ([EMIM][TFSI]) ion liquid, poly(ethylene glycol)diacrylate (PEGDA) monomers, and 2-hydroxy-2-methylpropiophenone (HOMPP) photo-initiator in a weight ratio of 90 : 7 : 3)^19^ were patterned on the PEDOT:PSS aligned with the Pt/Au electrode by photolithography, followed by kinking the active electrode (Ag electrode)-coated cotton fibers in a horizontal orientation. It is well known that the quality of the electrode is the prerequisite for an excellent performance of devices. Therefore, the metal-coated fibers are characterized firstly. Fig. S1† shows the scanning electron microscopy (SEM) images of Ag-coated (left panel) carbon fibers and Pt-coated (right panel) carbon fibers. The carbon fibers are commercially available (MSC KOREA, M1536, typical fiber diameter of 5–10 μm). As shown in Fig. S1† left panel, Ag particles are uniformly adhered to the fiber surface by thermal evaporation, forming a core-clad structure surrounding raw fibers. In previous iontroinc system-based memristor neural networks device or neuromorphic memory devices, the mechanisms involved were trap-de-trap mechanism,^24^ ion-intercalation mechanism,^25,26^ phase mechanism,^27^ and ion control mechanism^28,29^

Fig. 1c systematically illustrates the mechanism of the fiber-type iontronic memristor. Initially, no voltage was posed on the device. The ions in the ion gel turn out a random distribution with cations and anions fully balanced with each other. Upon the positive voltage exerted on the device, the anions aligned along the top surface of the ion gel, inducing an electrical double layer (EDL) near the surface of the ion gel, leaving cations at the ion-gel/PEDOT interface, which was equivalent to applying a positive voltage on the semiconductor channel and trigger the conductivity, being referred as the “writing” process. As the positive voltage generally decreases, the EDL undergoes downsize (implying the decrease in the amounts of cation and anion), and cations at the ion-gel/PEDOT interface decrease. As a consequence, the positive field control weakens on the semiconductor channel, and the conductivity decreases, returning to a high resistance state, which can be recognized as an “erasing” process. As the negative voltage is exerted on the device, the cations aligned along the top surface of the ion gel, inducing an electrical double layer (EDL) within the ion gel, leaving anions at the ion-gel/PEDOT interface, which was equivalent to applying a negative voltage on the semiconductor channel and trigger the conductivity, being referred as the writing process. As the negative voltage generally decreases, the EDL undergoes downsize (implying that the amounts of cation and anion decrease), and cations at the ion-gel/PEDOT interface decrease. As a consequence, the negative field control weakens on the semiconductor channel and the conductivity decreases, returning to a high resistance state, which can be recognized as an erasing process.

### 2.2 Electrical performance of the single unit of the memristor

**Fig. 2:**
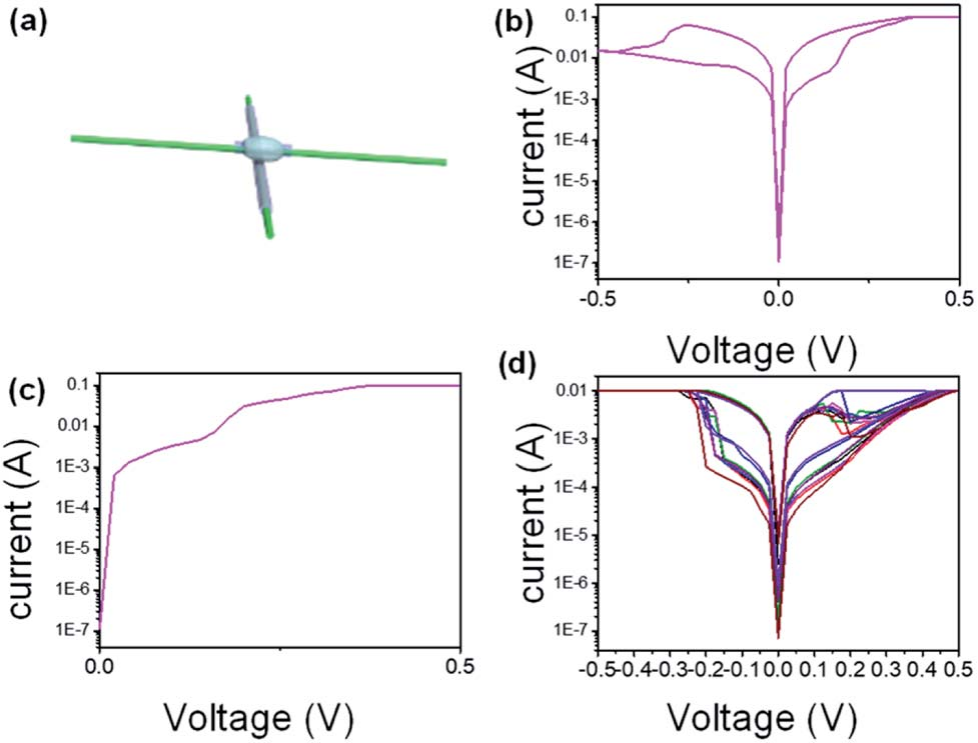
Memory device characteristics. (a) Schematic illustration of a single device in integrated memory-sensing system memristor array. (b) The typical *I*–*V* curve of the device under Icc of 100 μA (c) and 500 μA. Flexible TS device with a super nonlinearity of ≈106 at VD = 0.5 V and a fast turn-on switching slope of <4 mV dec−1. (c) The typical *I*–*V* curve of the device under varied Icc of 0.1 mA, 0.05 mA, 0.01 mA, and 0.005 mA (d) Consecutive 150 cycles of DC voltage sweeps with CC of 100 μA show no deterioration. Positive and negative voltage sweeps were alternately applied.

Prior to investigating the properties of the memristor matrix array, the electrical performance of the single unit was firstly explored. Fig. 2a displays the schematic illustration of a single memristor device. Similar to the aforementioned configuration memristor matrix array, the single unit also adopts a MIM configuration (semiconductor layer-PEDOT:PSS and dielectric layer ion-gel sandwiched between an active electrode and an inert electrode). As shown in Fig. 2b, a high initial OFF resistance state (10^7^ Ω) requires an electroforming process with the application of a forming voltage around 0.05 V to activate the bidirectional RS behaviour. The maximum value of nonlinearity is 10^7^ under the voltage of 0.5 V and a steep variation in the resistance from the initial high-resistance state to a low-resistance state, only needing a small positive voltage of about 0.02 V. In particular, the threshold voltage to open the device is much lower (could operate at a low gate voltage) due to the ultrahigh capacitance of the ion gel gate (6–7 μF cm^−2^), which considerably decreases the power consumption. The power consumption is about 5 × 10^−8^ J. The relatively lower operation voltage stems from the extremely high capacitance of the ion gel as the dielectric material^17–19,30^ and considerably decreases energy consumption. This also demonstrates the iontronic system in the development of electronics technology with low power consumption. The device shows bipolar resistor changing behaviour due to the bipolar transport characteristic of the semiconductor PEDOT. To validate the bidirectional TS behaviour, the sweep path of 0 → −0.5–5 → 0 → 0.5–5 → 0 V was operated on the device. Fig. SI2† illustrates the back-forward sweep of the device with no pronounced variation, indicating that the output is independent of voltage polarity.

To illustrate the role of the ion gel in the device, the device with Pt electrode/PEDOT/Ag electrode structure but in the absence of ion gel is measured as a comparative experiment. As shown in Fig. SI3,† the device in the absence of ion gel turns out to be of higher conductivity but has little hysteresis. This can be explained by the fact that the device with Pt electrode/PEDOT:PSS/Ag electrode structure in the absence of ion gel shows the property of the semiconductor of PEDOT:PSS. It is well known that the content of ion liquid in the ion gel has something to do with the ion regulation properties. Thus, the devices applied with different concentrations of ion liquid in ion gel were prepared, and the paralleled measurement was carried out. Fig. SI4† presents the typical *I*–*V* curve of device under varied ion gel recipes composed of 1-ethyl-3-methylimidazolium bis(trifluoromethyl sulfonyl)imide ([EMIM][TFSI]) ion liquid, poly(ethylene glycol)diacrylate (PEGDA) monomers, and 2-hydroxy-2-methylpropiophenone (HOMPP) photo-initiator in a weight ratio of 1.72 : 0.4 : 0.12 (pink curve) and 4.5 : 0.4 : 0.12 (blue curve). This is clearly shown in Fig. SI4,† with a higher amount of ion liquid of [EMIM][TFSI], and the window is larger but has lower cycling stability.

The device behaviour under a pulse voltage also can be served as a characteristic index for reflecting the relaxation properties. The pulse voltage posed on the device and the corresponding *I*–*t* curve is displayed in Fig. SI5† (pulse width 20 us, period 1 ms). The blue line: 20 mV pulse voltage and corresponding to high resistance state (HRS) red line: 500 mV pulse voltage and corresponding to the low resistance state (LRS). Whatever the high resistance state (HRS) and the low resistance state (LRS), the retention time can last 500 us, demonstrating good retention behaviour. The relaxation characteristics of the flexible TS device are sufficient for most flexible functional sensory elements, which typically show a relaxation time from 1 ms to 10 s.^31,32^

It is widely known that the complement current is also an important parameter to determine the performance of the device. Fig. SI6† displayed the typical *I*–*V* curve of the device under varied complement currents of 0.1 mA, 0.05 mA, 0.01 mA, and 0.005 mA. The ON/OFF is almost the same as varied complement currents but with sacrificing the hysteresis. Based on the evolution of current–voltage (*I*–*V*) characteristics as a function of complement currents, the maximum allowed complement currents for reliable operation for devices are about 10^−3^ A. Fig. SI 7† illustrates the typical *I*–*V* curves of the device under a varied sweep range (−1–1 V, −0.8–0.8 V, −0.6–0.6 V, −0.4–0.4 V), the different shapes of these curves demonstrate that this device can tolerate voltages with a large range.

Sufficient cycle stability is of paramount importance in a memristor device. As shown in Fig. 2d, the dark curve, red curve, blue curve, and purple curve illustrate the *I*–*V* performance of the memory device in the initial cycle, 10 cycles, 20 cycles, and 200 cycles. The rough overlap of every curve demonstrates its pronounced endurability. The device can withstand an endurance of over 200 cycles under the 100 μs/2 V programming pulses and 100 μs/0.3 V read pulses, with a 1.8 ms wait time between each program and read pulse (As shown in Fig. SI8†).

The device also possesses excellent environmental stability. Fig. SI9† shows the typical *I*–*V* curve of a device in a pristine state (blank line) and for four months (blue line). It can be clearly observed that the properties of the as-prepared device are almost identical to those of the pristine state and stayed in an ambient environment for four months later.

### 2.3 The sensing performance of the single unit of the memristor

**Fig. 3:**
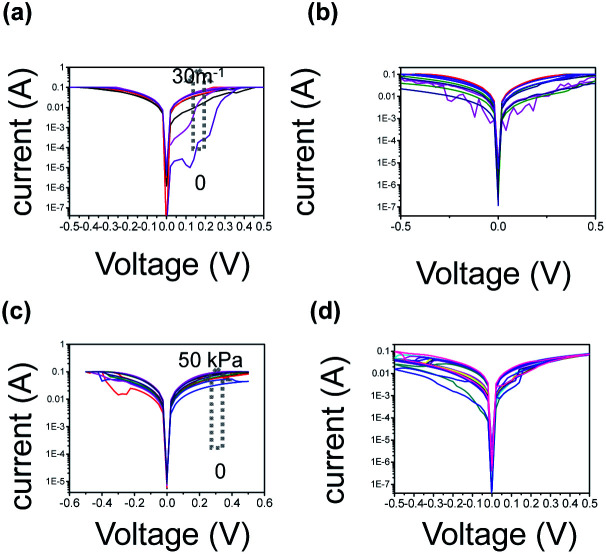
The memristor device characteristics under deformation (a) The typical *I*–*V* curve of device under varied bending degrees (curvature radius 12.7 m^−1^, 17.9 m^−1^, 21.8 m^−1^, 28.0 m^−1^ and 38.7 m^−1^). (b) The cycle *I*–*V* curve of device under a bending degree of 38.71 cm^−1^. (c) The typical *I*–*V* curve of the device under varied pressure degrees. (d) The cycle *I*–*V* curve of device under pressure degree of 25 kPa.

As aforementioned in the pretext, the iontronic system endows the device with stimuli-responsiveness. The characteristics of the integrated memory-sensing device under deformation have been systematically investigated. Fig. 3a shows the typical *I*–*V* curves of the device under varied bending degrees (curvature of 12.7 m^−1^, 17.9 m^−1^, 21.8 m^−1^, 28.0 m^−1^ and 38.7 m^−1^). It can be seen in Fig. 3a that as the bending degree increases from 12.7 m^−1^ to 38.7 m^−1^, the current increases from 13.8μA to 6820μA at a given voltage of 0.1 V, and the corresponding resistance decreases from 7246 Ω to 1.47 × 10^−5^ Ω. The higher bending degree leads to higher conductivity (relatively low resistance). This can be explained by the fact that as the device is in the bending state, the capacitance of the ion-gel increases and leads to an increase in conductivity. Fig. 3b depicts the endurability of the device in the bending state, that is, the cycle *I*–*V* curve of the device (*I*–*V* performance of the memory device in the initial cycle, 10 cycles, 20 cycles, and 200 cycles) under a bending degree of 38.71 cm^−1^. The device shows excellent bending flexibility without sacrificing cycle stability. The stability of the device in a repetitive bending state indicated its promising flexible wearable applications. Apart from bending deformation, compressing deformation is also studied. The typical *I*–*V* curve of the device under varied pressure (20 kPa, 40 kPa, 60 kPa, 80 kPa) is shown in Fig. 3c. The pressure deformation confirms a similar law: a higher compressing degree leads to higher conductivity (low resistance). To be specific, as pressure increases from 20 kPa to 80 kPa, the current elevated from 12090μA to 36230μA at a given voltage of 0.1 V, that is the corresponding resistance decreased from 8.27 × 10^−4^ Ω to 2.76 × 10^−4^ Ω. Similar to the pretext bending deformation, this can also be ascribed to the fact that as the device is in a loaded state, the capacitance of ion-gel increase and lead to an increase of conductivity. Fig. 3d depicts the *I*–*V* curve of the device under varied pressure. The almost overlap of curves at different pressure indicates its superior endurability in the compressing state (under the loaded state of 50 kPa). The device shows excellent bending flexibility without sacrificing cycle stability. Fig. S10† illustrates the typical *I*–*V* curve of the device with free-standing state, compressing state (50 kPa), and recovery state after experiencing pressure. As shown in Fig. S10,† the resistance value is restored when it returns to the flat state, providing the basis for applying in practical clothes or other wearable electronics. The maintained properties of the memristor device in deformation states originated from the conformability of the ion gel on the cotton wires and after repeatedly in the cycles of flat state-bending state alternating change. It was worth mentioning that ion gel guaranteed the memristor devices conformaly contact without losing the intimate contact property.

### 2.4 The mechanism of the memristor

**Fig. 4:**
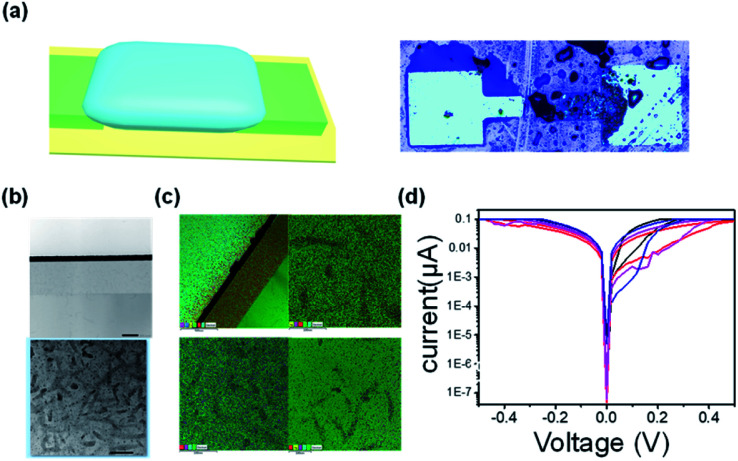
Memristive mechanism analysis based on a coplanar structure (a) schematic illustration (left panel) and an optical microscope image (right panel) of the coplanar structure (b) low-magnification transmission electron microscope TEM image corresponding to the initial off-state. Scale bar 20 nm. (c) EDS spectrum taken in the framed area in (d) typical *I*–*V* curve of device with different areas.

However, the EDLT produced by ion gel has dual functions of electrochemical doping and electrochemical dynamic. What mechanism plays the predominant role? Or whether both the Ag filament mechanism and electrochemical dynamic coexist? So, we constructed a device with the Au-EDLT-Pt (in the absence of Ag filament-mechanism) for a comparison. If the properties of the device distinguish from the counterpart of Ag-EDLT-Pt, the Ag filament mechanism may play a vital role. Otherwise, if the properties are the same as the counterpart of Au-EDLT-Pt, the mechanism is not based on Ag filament-mechanism. To get insight into the mechanism, the device to be studied adopted a coplanar structure. Fig. 4a shows the schematic illustration (left panel) and an optical microscope image (right panel) of the device in coplanar structure. The details of the fabrication process are depicted in the Experimental Section. In a previous work, it has been verified that woven devices can work reliably even though the interfaces between the two fibers are not completely adhered.^33^ The wearable electronic textiles are subjected to extensive mechanical stress and deformation, which might induce the separation or the shift of contact position between threads. With respect to the mechanism of the typical Ag-based (Ag electrode as the active electrode) two-terminal memristor, Ag filament-mechanism is relatively common.^34–37^ However, due to the thickness of the ion gel reaching 100 μm, the Ag conductive filaments are not able to diffract to the counter electrode. We anticipated that the mechanism of this device was not Ag conductive filament mechanism. To verify our hypothesis, TEM is applied.

The transmission electron microscope (TEM) is a powerful tool for investigating the mechanism of memristor devices. A positive bias pulse poses on the as-prepared device to trigger the device to its on-state. The TEM is carried out as the device is kept in its on-state. As shown in Fig. 4b, different from the typical memristor with a conductive filament mechanism, the crystal does not grow from the active electrode base and touches the inertia electrode and does't forming a triangle-like morphological domain. We carried out the nanoprobe energy-dispersive X-ray spectroscopy (EDS) maps in four different regions within the ion gel, and there is no Ag element signals observed in all regions (Fig. 4c). It provided additional evidence to verify that the mechanism of this device was not based on Ag conductive filament mechanism. To further solidify this standpoint, we compare different devices having various areas. As shown in Fig. 4d, the magnitude of the hysteresis and ON/OFF of different devices with various areas (100 μm^2^, 400 μm^2^, 1000 μm^2^) are nearly the same, demonstrating that the mechanism of this device was not Ag conductive filament mechanism, which was strongly associated with the area of the electrode.^38^ To further demonstrate the non-Ag-conductive filament mechanism, the Au electrode replaced the Ag electrode to obtain the device Pt/PEDOT:PSS-ion gel/Au as a comparative experiment. The typical *I*–*V* curve of the device Pt/PEDOT:PSS-ion gel/Au is shown in Fig. SI11.† It is clearly observed that the performance of the device Pt/PEDOT:PSS-ion gel/Au is almost identical to the counterpart: Pt/PEDOT:PSS-ion gel/Ag, verifying that the mechanism is not associated with the metal conductive filament mechanism (due to the inertia of Au and difficult to produce metal conductive filament mechanism).

To demonstrate the universality of this fiber-type memristor, the P3HT was used to replace PEDOT as an active material. The typical *I*–*V* curve of the device with P3HT as active material of memristor under varied sweep range is shown in Fig. S12† (−1–1 V, −0.8–0.8 V, −0.6–0.6 V, −0.4–0.4 V) As shown in Fig. S12,† the device also possesses a high ION/I OFF (10^6^) and low-voltage operation.

### 2.5 The memristor array

**Fig. 5:**
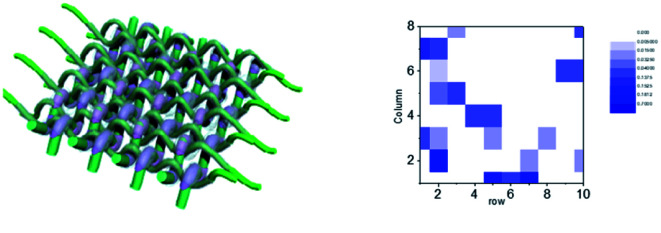
The integrated sensing-memory fiber-type memristor array. (a) The schematic diaikgram memristor array. (b) Pressure 2D mapping of the memristor array.

Finally, the aforementioned integrated sensing-memory fiber-type memristor was extended to an 8*8 array as sensory nervous systems. As shown in Fig. 5a, an integrated sensing-memory array was fabricated. Prior to conducting the sensation application, electrical performance of each memristor unit was measured. All the 64 units could perform well. The memristor array was attached on an immobilized vertical frame, and the target object attached on the opposite frame was controlled by a step motor to execute approaching the memristor array. The pressure could be reflected from the output signals of each unit of the array under the same voltage and plotted in 2D black-and-white coordinates (Fig. 5b). It was noteworthy that initializing the devices only needed a voltage of 0.1 V, which is very important in low-power-consuming sensation applications. Meanwhile, only the sensing pixel showed output signals according to the approaching process, indicating no crosstalk in the array.

## 3 Conclusions

In summary, we constructed an integrated fiber-based sensing-memory to achieve high-level integration and miniaturization. The integrated fiber-based sensing-memory is realized by the particular properties of the ion-gel system. Furthermore, the ion-gel system endows the device with low-operation characteristics and a considerable decrease in energy consumption. Meanwhile, the weaving-type memory array presents conformally attached skin. The device turns out to be of bipolar characteristic with an excellent ON/OFF (10^6^), good durability (200cycles), considerably surpassing the transitional PEDOT:PSS memristor device (ON/OFF < 10^3^, durability <50cycles). The relationship of resistance *vs.* pressure turns out to be relatively linear with excellent stability of output under deformation. Our work will provide new insight for the development of the sensing-memory neuromorphic network and significantly promote the chip to achieve high-level integration and miniaturization.

## 4 Experimental

### 4.1 Materials

The cotton fibres were bought from Hangzhou Yangyang cool company. PEDOT:PSS ink (≥99%), ion liquid 1-ethyl-3-methylimidazolium bis(trifluoromethyl sulfonyl)imide ([EMIM][TFSI]) (≥98%), poly(ethylene glycol)diacrylate (PEGDA) monomers (≥98%), and 2-hydroxy-2-methylpropiophenone (HOMPP) (≥99%) were bought from Admas chemical limited liability company.

### 4.2 Fabrication method

(1) Fiber-coated electrode fabrication.

The commercially-available cotton fibers in horizontal orientation selectively coated inertia electrode (*e.g.* Pt, Au electrode, 5 nm/70 nm, Cr/Au and 5 nm/90 nm Cr/Pt) with tightly adhered shadow mask through thermal evaporation methods or magnetron sputtering, followed by dip-coating with PEDOT:PSS ink (1 wt%). Prior to ion gel pattern, PEDOT:PSS ink coated fiber was annealed at 90–110 °C for 1 h.

Above the PEDOT:PSS corresponding to the position of inert electrode, ion gel (composed of 1-ethyl-3-methylimidazolium bis(trifluoromethyl sulfonyl)imide ([EMIM][TFSI]) ion liquid, poly(ethylene glycol) diacrylate (PEGDA) monomers, and 2-hydroxy-2-methylpropiophenone (HOMPP) photo-initiator in a weight ratio of 90 : 7 : 3) were patterned by photolithography (exposure time 12 s, 275 W), followed by knitting the active electrode (Ag electrode, 5 nm/70 nm Cr/Au)-coated cotton fiber in horizontal orientation.

(2) The coplanar structure for mechanism investigation through transmission electron microscope TEM.

Si substrate (120 μm) was cleaned under sonication with acetone, isopropanol, and deionized water (DI) water, respectively. The inertia electrodes (*e.g.* Pt, Au electrode, 5 nm/70 nm Cr/Au and 5 nm/90 nm Cr/Pt) were evaporated through electron beam evaporating 100 nm Ag and patterned *via* typical photolithography procedure (AZ 5214 as photoresist, exposure for 10 s under UV light of 275 W), followed by metal etching. Ag active electrode was patterned *via* typical photolithography procedure (AZ 5214 as photoresist, exposure for 10 s under UV light of 275 W) and followed by electron beam evaporating 100 nm Ag and lifting off procedure by acetone. PEDOT:PSS patterned by means of ink-jet printing of PEDOT:PSS ink (1%) between the inertia electrodes and active electrodes. The ion gel consisting of 1-ethyl-3-methylimidazolium bis(trifluoromethyl sulfonyl)imide ([EMIM][TFSI]) ion liquid, poly(ethylene glycol)diacrylate (PEGDA) monomer, and 2-hydroxy-2methylpropiophenone (HOMPP) photo-initiator (90 wt%: 7 wt%: 3 wt%) was cast above the patterned PEDOT:PSS. Under UV exposure, polymerization of PEGDA was initiated by the reaction between monomers and radicals originating from HOMPP to produce a cross-linked structure. The unexposed region was washed away using DI water.

Specially, prior to dip-coating the ion gel, rendering the surface of fibres exhibiting hydrophilicity/hydrophobicity in defined different regions was according to the previous work by Wu W. Q.^39^

### 4.3 Characterization

Surface morphology was observed under a SEM (FEI/Quanta 450 FEG). X-ray diffraction (XRD) patterns were obtained on a Panalytical X-ray diffractometer with Cu radiation of 2.2. The samples were scanned in the 2θ range of 10–80° with the step size of 0.05. Raman (HORIBA/LabRAM HR Evolution) measurements were performed on a RenishawInVia. Raman microscope was equipped with a 532 nm laser. The electrical performances of the memristor were performed on a Keysight B1500A semiconductor device analyzer. All measurements were carried out under ambient conditions.

## Author contributions

Yanfang Meng conceived the experiment. Yanfang Meng and Jiaxue Zhu conducted the experiments. Yanfang Meng analysed the results. All authors reviewed the manuscript. All figures were drawn by ourselves.

## Conflicts of interest

There are no conflicts to declare.

## Supplementary Material

NA-004-D1NA00703C-s001
