# Supplementary material for: Low energy consumption fiber-type memristor array with integrated sensing-memory
Source: Nanoscale Adv. 2022 Jan 18;4(4):1098–104. doi: 10.1039/d1na00703c (PMC9417447; doi:10.1039/d1na00703c)
Supplement: NA-004-D1NA00703C-s001 [file NA-004-D1NA00703C-s001.pdf]

Electronic Supplementary Material (ESI) for Nanoscale Advances.
This journal is © The Royal Society of Chemistry 2022

Supporting Information for

Low energy consumption fiber-type memresistor array with integrated sensing-memory

Yanfang Meng [*a,b,+] *Jiaxue Zhu* [c,+]

a. Department of Engineering Mechanics, Tsinghua University, Beijing, 100084, China.

b. Key Laboratory of Advanced Optical Communication Systems and network, School of electronic engineering and computer science department, Peking University.

c. Key Laboratory of Microelectronic Devices and Integrated Technology, Institute of Microelectronics of the Chinese Academy of Sciences, Beitucheng West Road, Beijing 100029,

Email: yanaimengmeng@126.com.

Supplementary Text

Figure S1. Scanning electron microscopy (SEM) images of Ag-coated (left panel) carbon fibers and Pt-coated (right panel) carbon fibers

Figure S2. The typical *I–V* curve of device back-forward sweep

Figure S3. The typical *I–V* curve of sole PEDOT: PSS(red line) and sole ion gel(blue line).

Figure S4. The typical *I–V* curve of device under varied ion gel recipe

Figure S5. The pulse voltage posed on the device and the corresponding *I-t* curve

Figure S6. The typical *I–V* curve of device under varied compliment currents

Figure S7. The typical *I–V* curve of device under varied sweep range (-1~1V, -0.8V~0.8V, -0.6V~0.6V, -0.4V~0.4V)

Figure S8. The endurance of over 200 cycles under the 100 μs/2 V programming pulses and 100 μs/0.3 V read pulses, with a 1.8 ms wait time between each program and read pulse.

Figure S9. The typical *I–V* curve of device Ag/PEDOT/Pt in fresh state (blank line) and for four months (blue line).

Figure S10. The typical *I–V* curve of device with free-standing state, compressing state (50 kPa) and recovery state after experiencing pressure.

Figure S11. The typical *I–V* curve of device with of memorisistor Pt/PEDOT:PSS-ion gel/Au (green line) and Pt/PEDOT:PSS-ion gel/Au(blank line)

Figure S12. The typical *I–V* curve of device with P3HT as active material of memorisistor under varied sweep range (-1～1V, -0.8V～0.8V, -0.6V～0.6V, -0.4V～0.4V).

Supplementary Figures

Figure S1

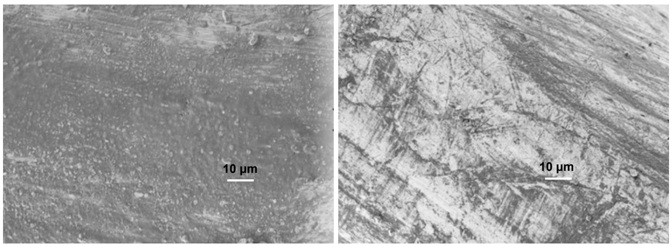

Figure S1. Scanning electron microscopy (SEM) images of Ag-coated (left panel) carbon fibers and Pt-coated (right panel) carbon fibers

Figure S2

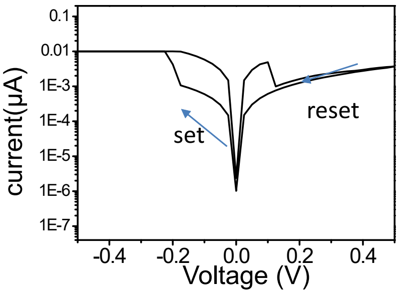

Figure S2. The typical *I–V* curve of device back-forward sweep

Figure S3

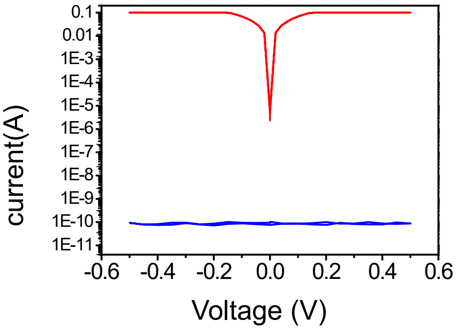

Figure S3. The typical *I–V* curve of sole PEDOT: PSS(red line) and sole ion gel(blue line).

Figure S4

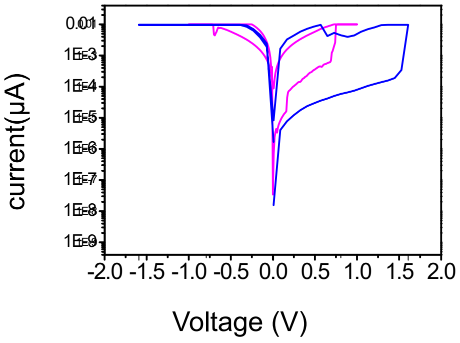

Figure S4. The typical *I–V* curve of device under varied ion gel recipe(composed of 1-ethyl-3-methylimidazolium bis(trifluoromethyl sulfonyl)imide ([EMIM][TFSI]) ion liquid, poly(ethylene glycol) diacrylate (PEGDA) monomers and 2-hydroxy-2-methylpropiophenone (HOMPP) photo-initiator in a weight ratio of 1.72:0.4:0.12 (pink curve) and 4.5:0.4:0.12(blue curve)

Figure S5

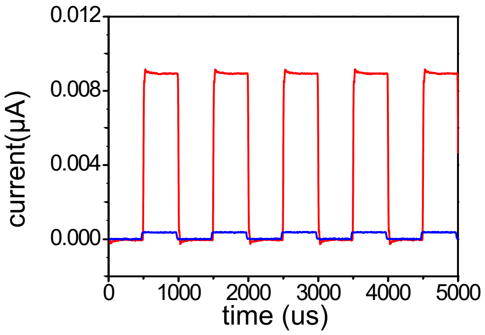

Figure S5. The pulse voltage posed on the device and the corresponding *I-t* curve

Figure S6

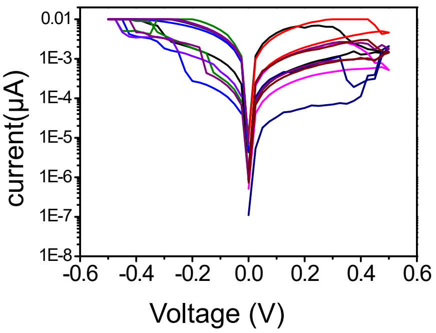

Figure S6. The typical *I–V* curve of device under varied compliment currents

Figure S7

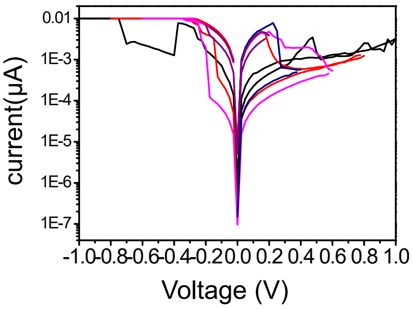

Figure S7. The typical *I–V* curve of device under varied sweep range (-1~1V, -0.8V~0.8V, -0.6V~0.6V, -0.4V~0.4V)

Figure S8

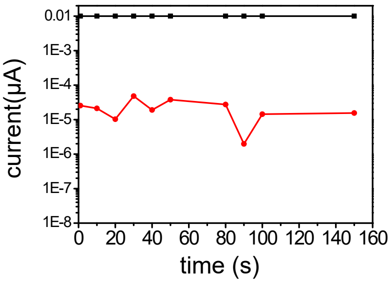

Figure S8. The endurance of over 200 cycles under the 100 μs/2 V programming pulses and 100 μs/0.3 V read pulses, with a 1.8 ms wait time between each program and read pulse.

Figure S9

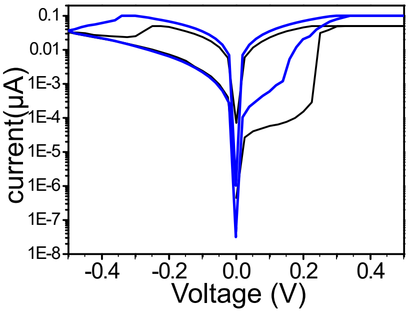

Figure S9. The typical *I–V* curve of device Ag/PEDOT/Pt in fresh state (blank line) and for four months (blue line).

Figure S10

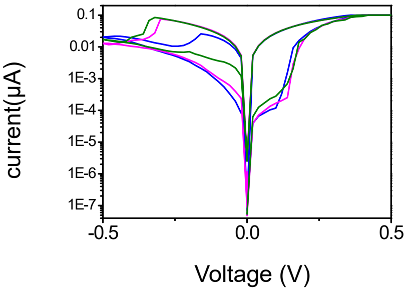

Figure S10. The typical *I–V* curve of device with free-standing state, compressing state (50 kPa) and recovery state after experiencing pressure.

Figure S11

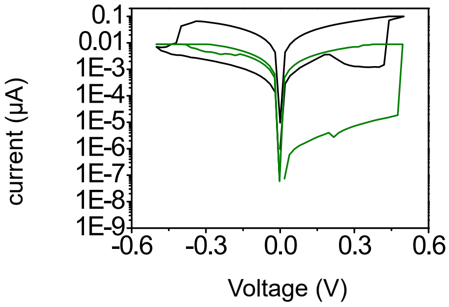

Figure S11. The typical *I–V* curve of device with of memorisistor Pt/PEDOT:PSS-ion gel/Au (green line) and Pt/PEDOT:PSS-ion gel/Au(blank line)

Figure S12

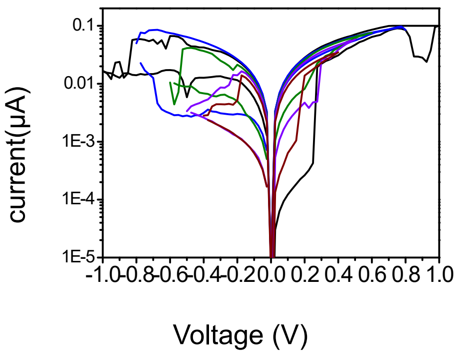

Figure S12. The typical *I–V* curve of device with P3HT as active material of memorisistor under varied sweep range (-1～1V, -0.8V～0.8V, -0.6V～0.6V, -0.4V～0.4V).
